# Structures of Angptl3 and Angptl4, modulators of triglyceride levels and coronary artery disease

**DOI:** 10.1038/s41598-018-25237-7

**Published:** 2018-04-30

**Authors:** Ekaterina Biterova, Mariam Esmaeeli, Heli I. Alanen, Mirva Saaranen, Lloyd W. Ruddock

**Affiliations:** 1 Faculty of Biochemistry and Molecular Biology and Biocenter Oulu, University of Oulu, Oulu, 90220 Finland; 2 Present Address: Department of Molecular Enzymology, University of Potsdam, 14476 Potsdam, Germany

## Abstract

Coronary artery disease is the most common cause of death globally and is linked to a number of risk factors including serum low density lipoprotein, high density lipoprotein, triglycerides and lipoprotein(a). Recently two proteins, angiopoietin-like protein 3 and 4, have emerged from genetic studies as being factors that significantly modulate plasma triglyceride levels and coronary artery disease. The exact function and mechanism of action of both proteins remains to be elucidated, however, mutations in these proteins results in up to 34% reduction in coronary artery disease and inhibition of function results in reduced plasma triglyceride levels. Here we report the crystal structures of the fibrinogen-like domains of both proteins. These structures offer new insights into the reported loss of function mutations, the mechanisms of action of the proteins and open up the possibility for the rational design of low molecular weight inhibitors for intervention in coronary artery disease.

## Introduction

Coronary artery disease (CAD) is the most common form of heart disease and is the most common cause of death globally, with CAD affecting 110 million people in 2015^1^. CAD is linked to a number of risk factors, including smoking^2^, diabetes^3^, family history^4^, stress^5^ and environmental factors^6^. Blood fats, including serum low density lipoprotein, high density lipoprotein, triglycerides and lipoprotein(a) are all strongly linked with CAD^7–14^.

Recently proprotein convertase subtilisin/kexin type 9 (PCSK9) inhibitors have been developed to inhibit LDL uptake^14,15^ and to reduce the incidence of CAD^16^. Parallel to this two other proteins have emerged – primarily from genetic studies – as being factors that significantly modulate plasma triglyceride levels and CAD. These proteins, human angiopoietin-like protein 3 (Angptl3; also known as Ang5) and angiopoietin-like protein 4 (Angptl4) are, as the names suggest, related structurally to angiopoietins which are involved in angiogenesis via their interaction with the Tie2 (tyrosine kinase with immunoglobulin and endothelial growth factor homology domain-2) receptor^17^. Both angiopoietins and angiopoietin-like proteins (except Angptl8) contain an N-terminal region, predicted to be intrinsically disordered, followed by a coiled-coil oligomerization domain and a C-terminal fibrinogen-like domain. While angiopoietins primarily retain all of these domains, Angptl4 and Angptl3 undergo proteolysis to release the functional fibrinogen-like domain^18–20^. The molecular mechanisms of action of these proteins are not as well defined, but functionally they appear to play a wider role than that of the angiopoietins. Angptl3 is primarily studied for its role in lipid and glucose metabolism^21,22^ and is involved in familial hypobetalipoproteinemia 2 (FHBL2), while Angptl4 has been reported to have wider but overlapping functions^23^ and has been implicated in wound repair^24,25^.

Recently there has been significant focus on both Angptl3 and Angptl4 being targets for CAD intervention, with Angptl3 being predicted to be the next PCSK9^26^. For Angptl3 loss of function mutations result in significantly lower levels of triglycerides, high density lipoprotein (HDL) cholesterol and low density lipoprotein (LDL) cholesterol^27–31^, with carrier status associated with a 34% reduction in CAD^29^. Inhibition of Angptl3 function in humans with the antibody evinacumab reduced triglycerides and LDL cholesterol levels in humans and decreased atherosclerotic lesion area and necrotic content in dyslipidemic mice^30^. The use of antisense oligos to Angptl3 in human trials significantly decreased triglycerides, LDL cholesterol, very low density lipoprotein (VLDL) cholesterol, non-high density lipoprotein cholesterol, apolipoprotein B and apolipoprotein CIII^32^. For Angptl4 loss of function mutations are reported to result in significantly lower triglyceride levels and higher HDL levels than among non-carriers and carriers were less likely to have CAD^27,33–36^.

While there is very significant overlap between the phenotypes associated with loss of function of Angptl3 and Angptl4 there are differences, for example Angptl3 loss of function decreases HDL cholesterol levels while loss of function of Angptl4 increases HDL cholesterol levels. The exact function and mechanisms of action of both proteins remain to be elucidated, but both have been reported to interact with lipoprotein lipase^37,38^.

Here we determined the structures of the fibrinogen-like domains of Angptl3 and Angptl4, structures which offer new insights into reported loss of function mutations, the mechanisms of action of the proteins and open up the possibility of rational design of low molecular weight inhibitors.

## Results and Discussion

### Protein production

**Figure 1:**
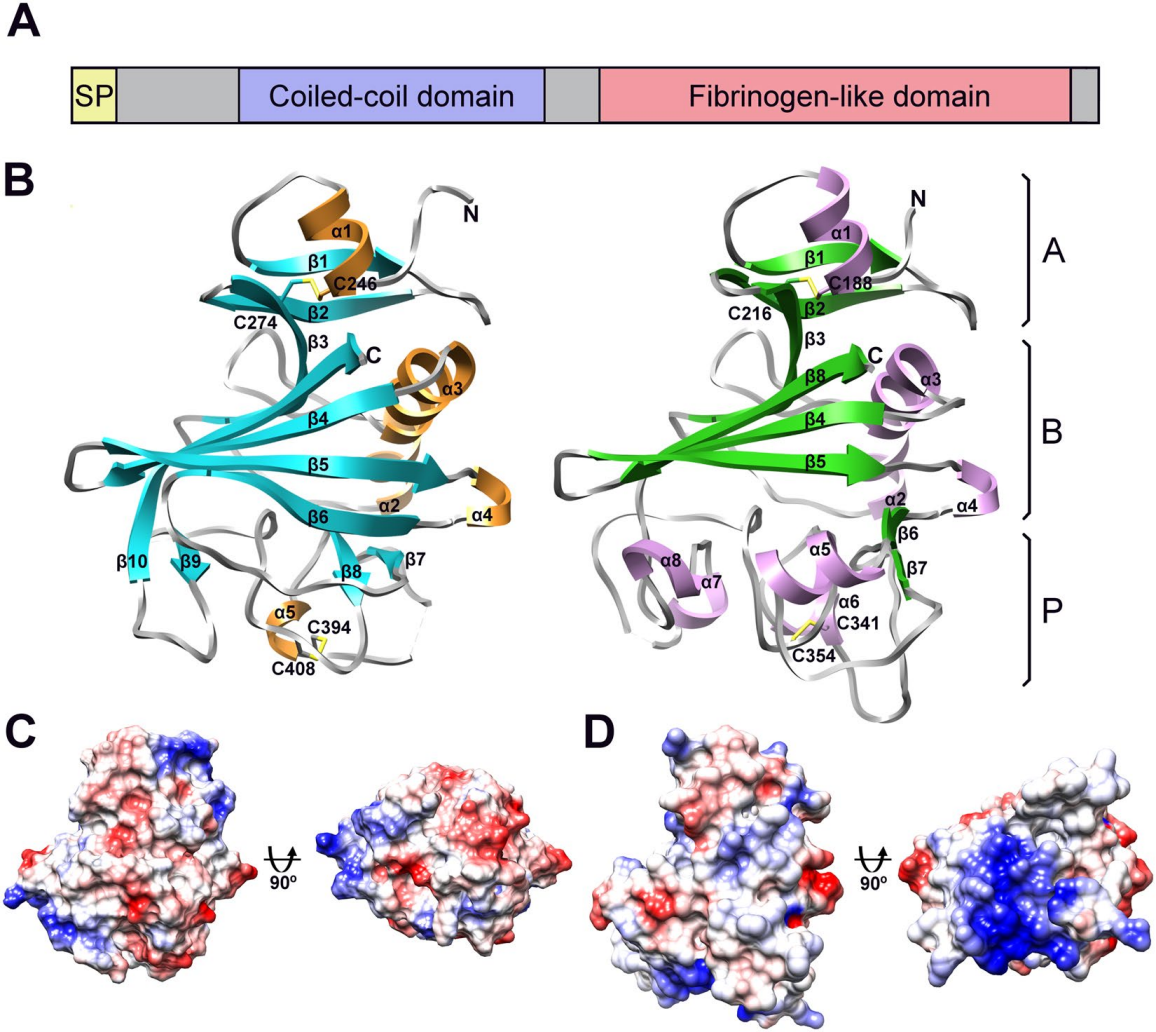
Crystal structure of the fibrinogen-like domains of Angptl3 and Angptl4. (**A**) Schematic of Angiopoietin-like protein domain architecture. Each angiopoietin and angiopoietin-like protein (except Angptl8) contains an N-terminal region predicted to be intrinsically disordered, a coiled-coil region and a C-terminal fibrinogen-like domain. (**B**) Structure of Angptl3 (left) and Angptl4 (right) fibrinogen-like domains. Each can be split into three subdomains A, B and P. Each structure is colored according to secondary structure elements with α-helices in gold (Angptl3) and violet (Angptl4), β-strands in cyan (Angptl4) and green (Angptl4) and loops in grey. Missing sections in the loops are indicated by dashed lines. Secondary structure elements are labeled and the N- and C-termini are indicated. (**C**) Electrostatic surface potential of Angptl3, the orientation is equivalent to that in panel B and rotated 90° around horizontal axis to see the underside of the P subdomain. Negative charge is shown in red and positive in blue. (**D**) As panel C but Angptl4.

Angiopoietins and Angiopoietin-like proteins (except Angptl8) have a similar domain architecture. After the signal peptide they have a region predicted to be intrinsically disordered followed by a coiled-coil domain and a C-terminal fibrinogen-like domain (Fig. 1A). Given their importance in CAD and emerging potential to serve as therapeutic drug targets modulating triglyceride levels in human we focused on angiopoietin-like proteins 3 and 4. Initial production trials for Angptl3 and Angptl4 were based on the co-expression of the folding factors Erv1p and PDI and the mature protein (Ser17-Glu460 and Gly26-Ser406 respectively) or for Angptl3, a construct which lacks part of the predicted N-terminal intrinsically disordered region (Phe37-Glu460). These constructs, as well as constructs that lacked the fibrinogen-like domain (Ser17-Gly241, Ser17-Thr225, Phe37-Gly241 and Phe27-Thr225 for Angptl3 and Gly26-Arg183 for Angptl4), were produced as inclusion bodies or in low amounts and/or were prone to proteolysis. Given that the fibrinogen-like domain in angiopoietins is the region which interacts with receptors and is reported to be released from Angptl3 and Angptl4 by proteolysis *in vivo*, focus switched to the functional C-terminal fibrinogen-like domains (Ile242-Glu460 and Leu184-Ser406 respectively). Both fibrinogen-like domains contain 4 cysteine residues, which by conservation with angiopoietins were thought to form two sequential disulfide bonds. Expression of the Angptl3 and Angptl4 fibrinogen-like domains with an N-terminal His-tag in the cytoplasm of *E*.*coli*, along with the folding factors Erv1p, PDI and cypB expressed from a second plasmid, resulted in the production of 80 mg/L of Angptl3 fibrinogen-like domain and 190 mg/L of Angptl4 fibrinogen-like domain.

Electrospray mass spectrometry of both proteins + /− treatment with N-ethylmaleimide indicated that both fibrinogen-like domains were of the expected mass and both contained two disulfide bonds.

### Structure determination of the fibrinogen-like domain of human Angptl3 and Angptl4

Crystals of the fibrinogen-like domains of Angptl3 and Angptl4 diffracted to 2.1 Å and 2.3 Å, respectively. The crystal structures were solved by molecular replacement and the models were refined to R/R_free_ values of 19.4/25.3 and 20/25.4 for Angptl3 and Angptl4, respectively. All data collection and refinement statistics are summarized in Table 1.

**Table 1:** Data collection and refinement statistics.

	Angptl3	Angptl4
PDB code	6EUA	6EUB
Data collection		
Wavelength (Å)	0.966	0.9999
Resolution range (Å)	43.68–2.1 (2.17–2.1)	47.16–2.3 (2.382–2.3)
Space group	P 21 21 21	C 2 2 21
Cell dimensions		
a, b, c (Å)	60.05 63.65 169.43	133.02 133.77 39.63
α, β, γ, (°)	90 90 90	90 90 90
Total reflections	148702	102775
Unique reflections	38793	16058
Multiplicity	3.8 (3.8)	6.4 (6.3)
Completeness (%)	99.30 (97.05)	98.88 (95.31)
Mean *I/ σ Ι*	7.14 (1.02)	8.45 (1.25)
R merge	0.1317 (1.206)	0.1492 (1.121)
R pim	0.07577 (0.6875)	0.06355 (0.4708)
CC_1/2_	0.993 (0.398)	0.995 (0.651)
Refinement		
R_work_ (%)	19.4 (27.2)	20 (31.6)
R_free_ (%)	25.3 (31.6)	25.4 (32)
Number of non-hydrogen atoms	5139	1790
macromolecules	4978	1731
ligands		16
solvent	161	43
Protein residues	601	216
RMS bonds (Å)	0.007	0.010
RMS angles (°)	0.79	1.06
Average B factor (Å^2^)	46.78	57.78
macromolecules	46.99	57.76
ligands		66.35
solvent	40.47	55.42

Statistics for the highest-resolution shell are shown in parentheses.

There are 3 monomers of Angptl3 in the asymmetric unit. The N-terminal His-tag as well as the C-terminal 6 amino acids are not visible. In addition, there are two regions within the fibrinogen-like domain (Lys389 - His391 and Pro420 - Arg429 in the A-chain) for which the electron density is either missing or not of sufficient quality to allow tracing of the chain, suggesting the presence of flexible loops.

The Angptl4 crystal contains 1 molecule per asymmetric unit and the visible polypeptide chain begins with Leu184 and ends with Met400, hence 6 amino acids of the C-terminus are not visible. There is one break in the mainchain of Angptl4 at Gln370, suggesting the loop that it is in is flexible.

### Structures of the fibrinogen-like domain of Angptl3 and Angptl4

The fibrinogen-like domains of Angptl3 and Angptl4 form compact structures and, according to fibrinogen nomenclature, can be divided to 3 subdomains A, B and P (Fig. 1B).

The N-terminal domain (subdomain A) consists of an α-helix (α1) and two β-strands (β1, β2) forming a small antiparallel sheet which is stabilized by a disulfide bond formed by Cys246 and Cys274 in Angptl3 and Cys188 and Cys216 in Angptl4. The architecture of subdomain A is the most conserved among the fibrinogen-like domain containing homologs and superimposes well in both Angptl3 and Angptl4.

Subdomain B is the largest subdomain in both molecules. In Angptl3, it is composed of five β-strands (β3, β4, β5, β6 and β10) forming a large twisted antiparallel β-sheet, and three α-helices (α2, α3 and α4). The β-sheet extends from one side of Angptl3 to another and forms a shallow water exposed crevice. The two helices (α2 and α3) are inserted between β3 and β4 on the opposite side of β-sheet and are connected by an extended loop. The short α-helix, α4, connects β-strands β5 and β6. β10 is a part of the C-terminus and packs between strands β3 and β4. The B subdomain of Angptl4 is similar to that of Angptl3; however, the extended β-sheet is formed by four antiparallel β-strands and instead of the long β-strand β6 in Angptl3, it contains 2 short β-strands (β6 and β7), which form an antiparallel β-hairpin and are connected via α-helix α5 and an extended solvent exposed loop.

The third subdomain (P) functions as a site for ligand binding. Consistent with this, the architecture and the sequence of subdomain (P) vary the most among fibrinogen-like domain containing proteins. In Angptl3, the P subdomain contains little regular secondary structure - three short β-strands (β7, β8 and β9) and a short α-helix (α5). β7 and β8 form a small antiparallel β-hairpin, which is braced together with α5 and stabilized by a disulfide bond between Cys394 and Cys408. The short β9 strand is followed by an extended solvent exposed loop and packs against the beginning of the β-strand β10. In contrast to Angptl3, the regular secondary structure of the P subdomain of Angptl4 is comprised of only three short α-helices (α6, α7 and α8). Similarly to Angptl3 it contains one disulfide bond (Cys341 and Cys354).

### Comparison to structures of fibrinogen-like domain containing homologs

**Figure 2:**
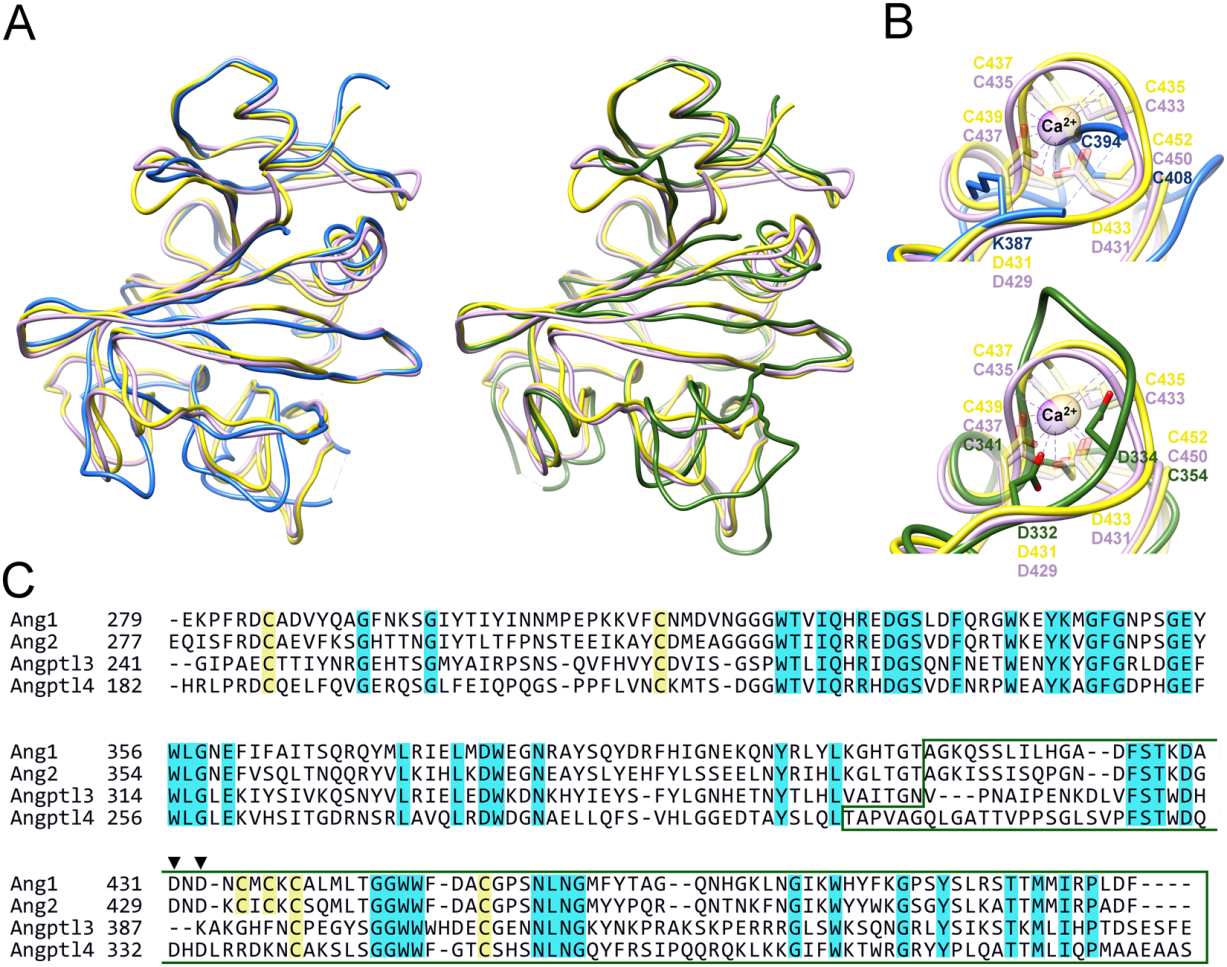
Comparison of the Angptl3 and Angptl4 fibrinogen-like domains with angiopoietins. (**A**) Overlay of the Cα-trace of Angptl3 (blue, left) and Angptl4 (green, right) with those of Ang1 (yellow) and Ang2 (violet). (**B**) Comparison with Ca^2+^ binding site of Ang1 (light yellow) and Ang2 (light violet) of the equivalent region in Angptl3 (blue, top) and Angptl4 (green, bottom). Residues involved in Ca^2+^ binding are shown in stick representation and colored similarly to the protein they belong. Ca^2+^ ions are shown in ball representation. (**C**) Alignment of the proteins shown in panel A. Cysteines are highlighted in yellow and conserved amino acids in blue. The P subdomain is boxed and the Asp involved in calcium binding in Ang1 and Ang2 are indicated with arrowheads.

The fibrinogen-like domains of human Angptl3 and Angptl4 share significant sequence and structural homology with other fibrinogen-like domains. Angptl3 can be superimposed on its closest structural homologues, identified by Dali server^39^, Angiopoietins 1 and 2 with a rms deviation of 2.3 Å (192 equivalent Cα positions; 4JYO^40^) or 2.4 Å (192 equivalent Cα positions; 1Z3S^41^) respectively. Similarly angiopoietins 1 and 2 are the closest structural homologs of Angptl4, with rms deviation of 2.7 Å (200 equivalent Cα positions) and 2.6 Å (200 equivalent Cα positions) respectively. Despite the poor sequence identity for subdomain A (Fig. 2A), for both proteins subdomains A and B superimpose well. In contrast, despite significant sequence identity, there are major structural differences among these proteins within the P subdomain, the subdomain involved in functional interactions and in the segments of the B subdomain immediately preceding P (Fig. 2). For example, the P subdomain of Angptl3 contains only 1 α-helical segment and 3 β-strands and does not superimpose well with structural homologs. In contrast, the P subdomain of Angptl4 is more comparable to those of structural homologs. However, the β-sheet of the B subdomain is narrower in Angptl4 than that of homologous structures and β-strand β6 is much shorter and turns away from the β-sheet towards the solvent and together with β7, α5 and an extended loop creates a separate solvent exposed element in the structure. DALI searches using just the P subdomain of Angptl3 or Angptl4 revealed no close structural homologs.

In contrast to Angiopoietin family members and other identified structural homologs, including M- and N-ficolins (PDB codes 2JHI and 2J2P;^42,43^) and tachylectin 5A (1JC9;^44^), which contain 3 conserved disulfide bonds, Angptls 3 and 4 are stabilized by only 2 disulfide bonds. The third pair of cysteines in Angiopoietins 1 and 2 (C435-C437 and C433-C435, respectively) forms a part of Ca^2+^ binding site along with two conserved aspartates (Fig. 2B). Calcium binding is required for fibrinogen polymerization^45^ and for agglutinating activity of tachylectin 5A^46^, however, Ca^2+^ binding site is not directly involved in Angiopoietin/Tie2 interactions but suggested to play a structural role by stabilizing coil elements involved in the interactions^41^. The two aspartates are conserved in Angptl4, but not in Angptl3 (Fig. 2C). No electron density, which would indicate the presence of Ca^2+^ or any other ion, was observed in either the Angptl3 or Angptl4 structures.

### Electrostatic surface potential

Analysis of the electrostatic surface potential of the fibrinogen-like domain of Angptl3 and Angptl4 revealed that the electrostatic charge is relatively evenly distributed over the majority of the surface of both molecules, except for the presence of an extended positively charged patch at the bottom of the P subdomain of Angptl4 (Fig. 1D), the surface that is most likely involved in the functional interactions. In contrast, no distinct electrostatic properties were identified on the surface of the P subdomain of Angptl3. This, when combined with other structural differences in the P domain, strongly suggests that Angptl3 and Angptl4 have different ligands and hence that they modulate triglyceride levels and CAD risk by distinct mechanisms. This is consistent with the observed difference that Angptl3 loss of function slightly decreases HDL cholesterol levels^30^ while loss of function of Angptl4 increases HDL cholesterol levels^33^.

### Loss of function mutations

A number of loss of function or reduction of function mutations have been reported for Angptl3 and Angptl4^27–36^. Many of these are frameshifts or premature stop codons, but others result from single amino acid substitutions in the fibrinogen-like domain. Using the determined crystal structures it is possible to elucidate why these mutations cause loss of function.

**Figure 3:**
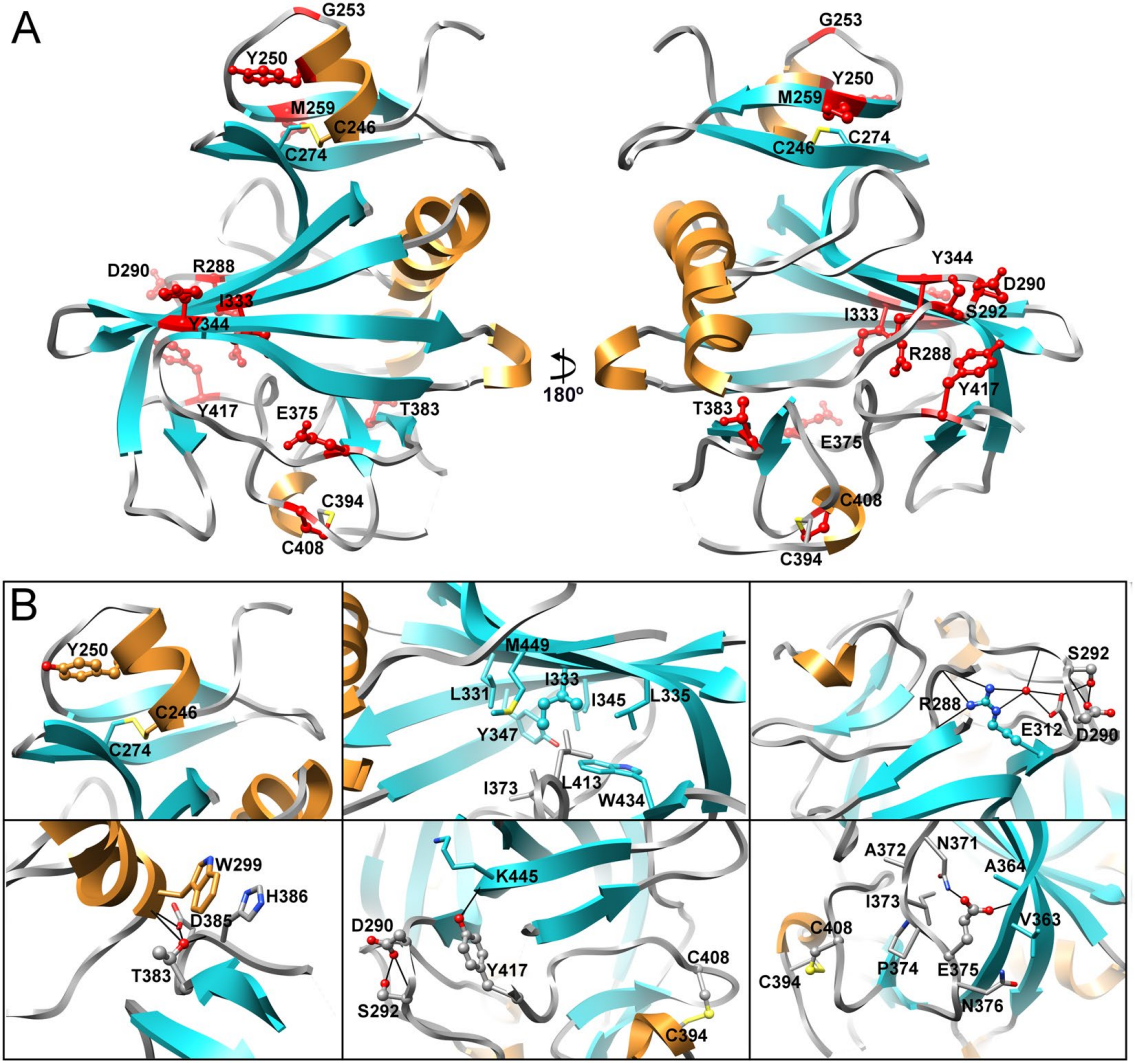
Structural analysis of mutations in Angptl3 which cause loss of function. (**A**) Angptl3 fibrinogen-like domain crystal structure with the sites of loss of function mutations depicted in ball and stick representation and highlighted in red. (**B**) Close up views of the environment of the mutated amino acids in panel A. Mutated residues are shown in ball and stick representation and neighboring or interacting residues are in stick representation. Residues are colored according to their location in the secondary structure elements with nitrogen atoms in blue, oxygen in red and sulfur in yellow. Potential hydrogen bonds are depicted as solid black lines.

For Angptl3 there are twelve reported point mutations in the fibrinogen-like domain which are associated with decreased triglyceride levels - M259T, R288Q, S292P, E375K and Y417C^27^, Y344S^31^ and, in decreasing order of severity, G253C, I333S, D290H, C408R, Y250C and T383S (;^29^ S292P also reported). These mutations are found in all three subdomains of Angptl3 (Fig. 3A). Four of the mutations, S292P, R288Q, E375K and Y417C abolish or severely reduce secretion *in cellulo*^27^ implying that they significantly affect folding and/or stability of Angptl3. The effects of all four can be explained from the structure by the disruption of structure stabilizing hydrogen bonding (Fig. 3B).

Potential structural defects associated with seven of the other eight mutations can similarly be elucidated using the solved crystal structure, either from potential effects on intra- or inter-molecular disulfide bond formation (G253C, Y250C, C408R) and/or disruption of structure stabilizing sidechain or backbone hydrogen bonds (Y250C, D290H, I333S, T383S) and/or disruption of regular secondary structural elements (I333S, Y344S, T383S) (Fig. 3B).

The effect of one mutation cannot easily be explained from the structure obtained here. Met259 is located on β1 in subdomain A with its side chain facing the solvent (Fig. 3A). It is possible that either the M259T changes interactions with the N-terminal region of Angptl3 not included in this structure or another undescribed interaction or influences cleavage to release the fibrinogen-like domain.

**Figure 4:**
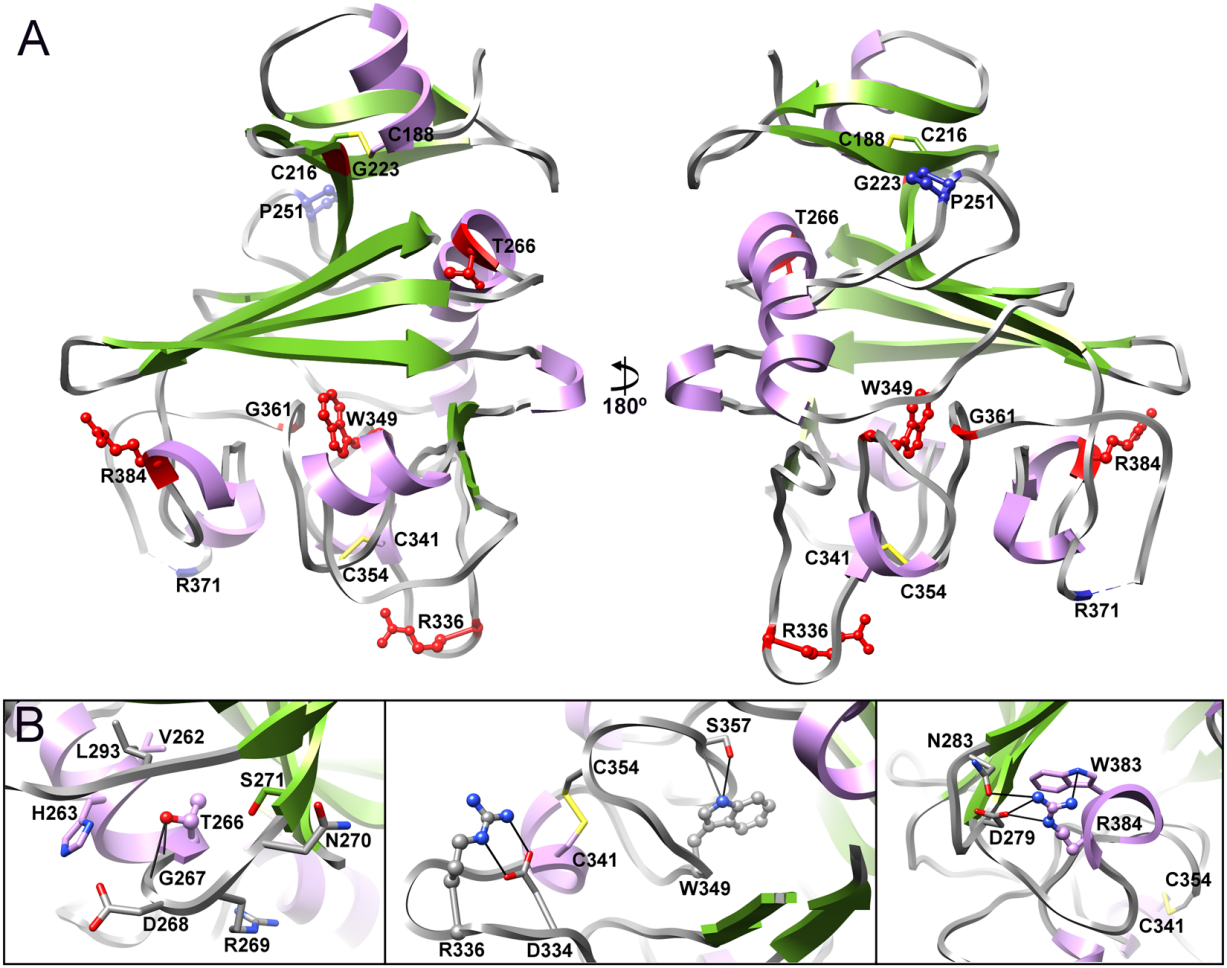
Structural analysis of mutations in Angptl4 which cause loss of function. (**A**) Angptl4 fibrinogen-like domain crystal structure with the sites of mutations resulting in lower triglyceride levels are indicated in red, mutations leading to the increase of triglyceride levels are shown in blue. (**B**) Close up views of the environment of the mutated amino acids in panel A. Residues are depicted and colored as in Fig. 3.

For Angptl4 there are six point mutations in the fibrinogen-like domain which are linked to decreased triglyceride levels - G223R, T266M, R336C, W349C, G361S and R384W (Fig. 4A^27,33,36^). Of these five (G223R, R336C, W349C, G361S and R384W) have been reported to abolish secretion *in cellulo*^27^ implying they effect the folding and/or stability of Angptl4. From the crystal structure all six might be predicted to either result in misfolding of the protein or to significantly destabilize the native state (Fig. 4B), by altering intra- or inter-molecular disulfide bonds (R336C, W349C) and/or disrupting structure stabilizing hydrogen bonds (T266M, R336C, W349C, R384W) and/or introducing steric clashes (G233R, T266M, G361S) and/or disruption of regular secondary structural elements (G361S).

For Angptl4 there are two reported mutations (P251T and R371Q) which are linked to increased triglyceride levels^33^. How these mutations increase triglyceride levels cannot easily be deduced from the structure. Pro251 is located in the middle of a solvent exposed loop between α2 and α3. The P251T mutation is unlikely to directly cause large conformational changes in this region of the protein. Thr has a higher propensity to be in regular secondary structure, especially β-sheet, than Pro and it is possible that the P251T mutation influences local structure and this is transmitted to subdomain P or that it increases the stability of the protein. Arg371 is located in the flexible loop after helix α7. This residue is not clearly visible in the electron density map and hence it is difficult to unambiguously suggest how the R371Q mutation increases triglyceride levels. Given the location of Gln370, Arg371 probably forms part of the positively charged patch of the surface at the bottom of the subdomain P which – by analogy with the angiopoietins – is probably involved in protein-protein interactions. Hence this mutation most likely directly modulates function rather than structure.

**Figure 5:**
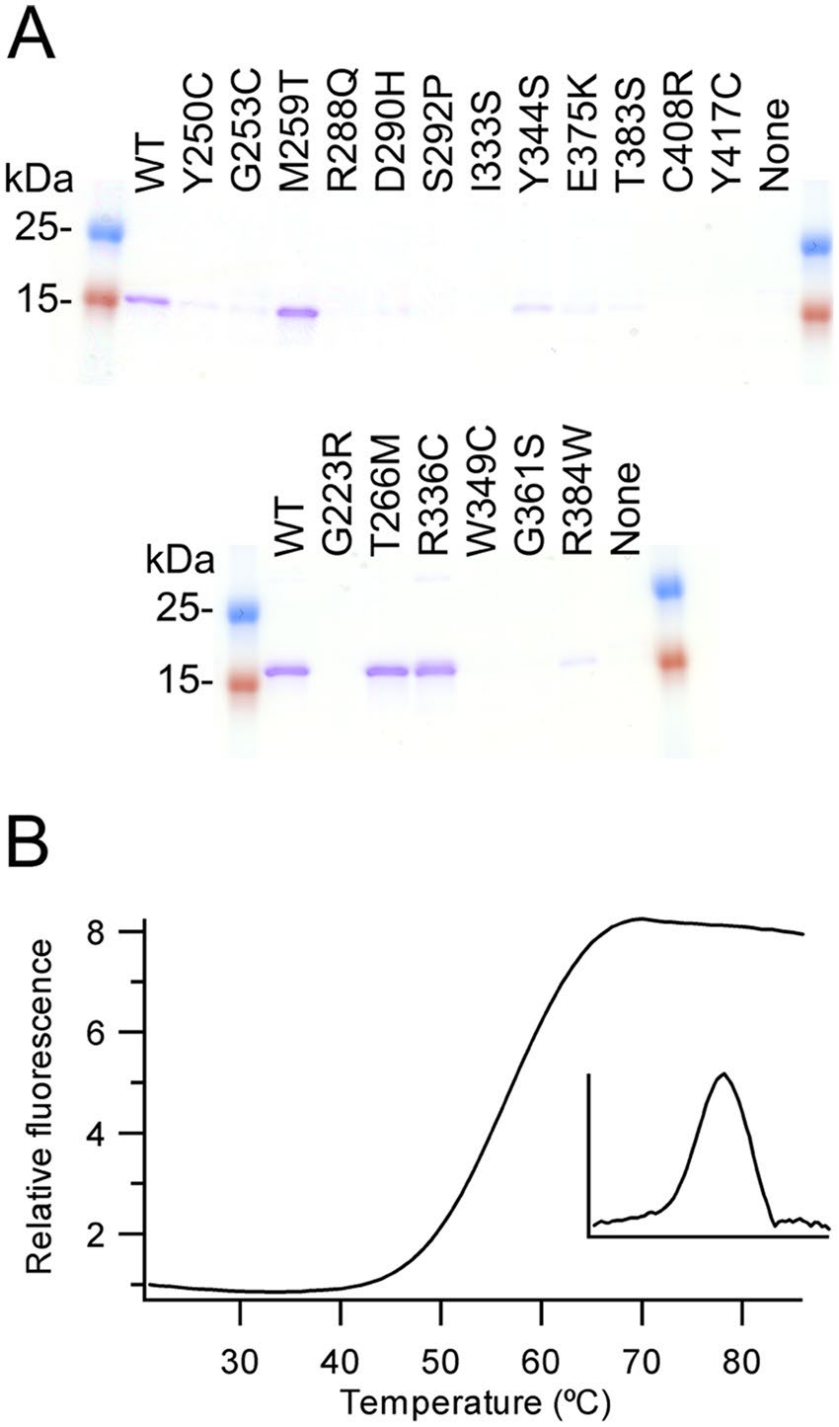
Analysis of loss of function mutations (**A**) Coomassie stained SDS-PAGE gel of IMAC purified proteins. Upper gel Angptl3, lower gel Angptl4 (Supplementary Fig. 1 shows the full gels) (**B**) Thermal stability of purified wild-type Angptl3 determined by thermofluor. The insert panel shows the derivative of the change in fluorescence signal. Angptl3 shows cooperative unfolding.

To further examine the potential effects on folding and/or structure of the mutations linked to a decrease in triglyceride levels, twelve mutations were made in Angptl3 and six in Angptl4 and the proteins expressed in the *E*.*coli* expression system alongside the wild-type proteins. Of the twelve mutations in Angptl3, eight (Y250C, R288Q, S292P, I333S, E375K, T383S, Y417C and C408R) resulted in no soluble protein being produced, while three (G253C, D290H and Y344S) resulted in greatly reduced yields compared with the wild-type protein (Fig. 5A). The one mutation which did not reduce the yield of protein made, M259T, was the one mutation we had been unable to assign a loss of function mutation based on the structure. To further analyze the effects of the mutation we examined thermal stability of the proteins. Wild-type and M259T mutant showed a single unfolding transition with a mid-point of 56.5 + /− 0.5 °C for wild-type and 57.7 + /− 1.1 °C for the M259T (Fig. 5B). Since the mutant again behaved like wild-type we can only assume that the M259T mutation changes interactions either with the N-terminal region of Angptl3 not included in this structure or another undescribed interaction.

Of the six mutants made in Angptl4, three gave no soluble protein (G223R, W349C and G361S) while one resulted in greatly reduced protein yields (R384W) (Fig. 5A). The remaining two (T266M and R336C) gave yields around those of the wild-type protein. The effects of the R336C mutation were unexpected as this mutation has previously been reported to abolish secretion *in cellulo*^27^. This difference may arise due to R336 being surface exposed and hence a cysteine at this position may result in the endoplasmic reticulum exit site machinery perceiving it as being non-native and hence retained. To further analyse the effects of the mutations we examined thermal stability. Wild-type and both mutants showed a single unfolding transition with the mid-point for R336C (62.0 + /− 0.0 °C), being higher than either the wild-type or T266M mutant (both 58.0 + /− 1.0 °C). As per the M259T mutation in Angptl3, the effects of the T266M mutation in Angptl4 on modulating triglyceride levels cannot be explained by structural effects. It is noteworthy that both lie in the A subdomain and hence the T266M mutation may also change interactions either with the N-terminal region of Angptl4 not included in this structure or with another as yet undescribed protein.

## Conclusions

The structures of the fibrinogen-like domains of Angptl3 and Angptl4 give new insights into these important proteins strongly linked to CAD, as well as offering the opportunity for the rational design of low molecular weight inhibitors of their function. In addition, the striking differences in the structures of subdomain P of the two proteins strongly suggests that while loss of function mutations of both lower plasma triglyceride levels, they act by different pathways.

## Materials and Methods

### Strains and expression constructs

All molecular biology in this study was performed using the *E*.*coli* strain XL1 Blue (*recA1 endA1 gyrA96 thi-1 hsdR17 supE44 relA1 lac F´[proAB lacI lacZ∆M15 Tn10 Tet*^*r*^]; Agilent Technologies), while protein production was performed in the strain MDS42 (multiple deletant strain;^47^).

Expression vectors (Table 2) were made by standard molecular biology techniques. Proteins of interest were cloned into a pET-derivative^48^ which contains an N-terminal hexahistidine tag (MHHHHHM-) for the protein of interest when cloned in using an in-frame 5′ NdeI restriction site and where the T7 promoter has been replaced with Ptac. *E*.*coli* codon optimized genes for mature human Angptl3 and Angptl4 were obtained from Genscript and these were used as templates for all domain constructs. The gene for mature human CypB was amplified from IMAGE clone 3502055 and the internal XbaI restriction site removed by mutagenesis (QuikChange; Agilent). CypB was then cloned into an existing CyDisCo variant pMJS205^48^ by SpeI/XhoI digestion of the vector and XbaI/XhoI digest of the insert. The resulting ligation gave a tricistronic vector (pME27) encoding Erv1p, PDI and CypB from a Ptac promoter. Plasmid purification was performed using the QIAprep spin miniprepkit (Qiagen) and purification from agarose gels was performed using the gel extraction kit (Qiagen), both according to the manufacturer’s instructions. The plasmids generated were sequenced (Biocenter Oulu core facility) to ensure there were no errors in the cloned gene.

**Table 2:** Plasmids used in this study.

Construct	Plasmid	Selection
Folding factors: Erv1p, PDI and CypB	pME27	Cm^R^
Angptl3 MH_6_M-S17-E460	pME19	Amp^R^
Angptl3 MH_6_M-F37-E460	pME20	Amp^R^
Angptl3 MH_6_M-S17-G241	pME21	Amp^R^
Angptl3 MH_6_M-S17-T225	pME16	Amp^R^
Angptl3 MH_6_M-F37-G241	pME22	Amp^R^
Angptl3 MH_6_M-F37-T225	pME17	Amp^R^
Angptl3 MH_6_M-I242-E460	pME23	Amp^R^
Angptl4 MH_6_M-G26-S406	pME24	Amp^R^
Angptl4 MH_6_M-G26-R183	pME25	Amp^R^
Angptl4 MH_6_M-L184-S406	pME26	Amp^R^
Angptl3 MH_6_M-I242-E460, Y250C	pMJS362	Amp^R^
Angptl3 MH_6_M-I242-E460, G253C	pMJS363	Amp^R^
Angptl3 MH_6_M-I242-E460, M259T	pMJS364	Amp^R^
Angptl3 MH_6_M-I242-E460, R288Q	pMJS365	Amp^R^
Angptl3 MH_6_M-I242-E460, D290H	pMJS366	Amp^R^
Angptl3 MH_6_M-I242-E460, S292P	pMJS367	Amp^R^
Angptl3 MH_6_M-I242-E460, I333S	pMJS368	Amp^R^
Angptl3 MH_6_M-I242-E460, Y344S	pMJS369	Amp^R^
Angptl3 MH_6_M-I242-E460, E375K	pMJS370	Amp^R^
Angptl3 MH_6_M-I242-E460, T383S	pMJS371	Amp^R^
Angptl3 MH_6_M-I242-E460, C408R	pMJS372	Amp^R^
Angptl3 MH_6_M-I242-E460, Y417C	pMJS373	Amp^R^
Angptl4 MH_6_M-L184-S406, G223R	pMJS374	Amp^R^
Angptl4 MH_6_M-L184-S406, T266M	pMJS375	Amp^R^
Angptl4 MH_6_M-L184-S406, R336C	pMJS376	Amp^R^
Angptl4 MH_6_M-L184-S406, W349C	pMJS377	Amp^R^
Angptl4 MH_6_M-L184-S406, G361S	pMJS378	Amp^R^
Angptl4 MH_6_M-L184-S406, R384W	pMJS379	Amp^R^

Selection is indicated by Cm^R^ for chloramphenicol resistance and Amp^R^ for ampicillin resistance.

### Protein expression and purification

*E*. *coli* MDS42 strain transformed with a vector for a Angptl3/4 expression along with the pME27 vector to catalyse disulfide bond formation were streaked onto LB-agar plates containing 100 µg/mL of ampicillin and 35 µg/mL of chloramphenicol and incubated overnight at 37 °C for expression in EnPresso B media (Biosilta Ltd). A single colony was then inoculated into 2 mL of LB media plus 2% glucose containing appropriate antibiotics at 30 °C and 250 rpm for 6 hours. The glucose represses expression from the Ptac promoter used for both CyDisCo components and the proteins of interest. This pre-culture was used to inoculate at a 1:100 ratio the desired culture volume (from 25 mL to 250 mL) of EnPresso B media. When using Ultra Yield (BioSilta) baffled flasks (Thomson Instrument Company), 5 µL Antifoam 204 (Sigma) per 50 mL of the culture was added. The flask was sealed with an oxygen permeable membrane (Thomson Instrument Company). Cultures were grown for 16–18 hours at 30 °C and 250 rpm and then protein expression was induced by isopropyl β-D-thiogalactoside (IPTG) to a final concentration of 0.5 mM. Culturing was continued for 24 hours after induction. Cells were collected by centrifugation at 3220 × g for 20 minutes at 4 °C. The pellet was re-suspended in 50 mM sodium phosphate buffer pH 7.4 supplemented with protease inhibitor cocktail (Roche) in a volume equivalent to half the culture volume.

### Protein purification

All constructs expressed had a hexa-histidine tag at their N-terminus to allow purification with standard immobilized metal affinity chromatography (IMAC) using HisPur Cobalt Superflow Agarose resin (Thermofisher). For large scale purifications the following protocol was followed: Lysis was performed by sonication 6 times for 5 seconds at 30 second intervals on ice. The lysate was clarified by 30 minutes centrifugation at 23500 × g at 4 °C and then filtered through a 0.45 µm filter before being applied to a 5 mL IMAC column previously equilibrated with 50 mM sodium phosphate pH 7.5. The resin matrix and lysate were mixed by inversion for 1 hour to allow batch binding and then washed with 4 column volumes of 50 mM sodium phosphate pH 7.5 and 20 column volumes of 50 mM sodium phosphate, 300 mM NaCl, pH 7.5. A manual imidazole step gradient was applied using at least 8 column volumes of 5 then 10 then 20 mM imidazole in 50 mM sodium phosphate pH 7.5 to wash out nonspecifically bound proteins. Angptl3 fibrinogen-like domain was eluted from the column by 3 column volumes of 150 mM imidazole, 50 mM sodium phosphate pH 7.5 followed by 3 column volumes of 200 mM imidazole, 50 mM sodium phosphate pH 7.5. Angptl4 fibrinogen-like domain was eluted applying a manual gradient of imidazole of 3 column volumes of 150, 200, 250 and 500 mM imidazole, 50 mM sodium phosphate, pH 7.4. For both proteins fractions with faint bands of impurities or no visible impurities on 12.5% SDS-PAGE were pooled and taken forward for subsequent purification steps. Pooled fractions from the IMAC were buffer exchanged and concentrated using Amicon® Ultra centrifugal concentrators (Millipore). Size exclusion chromatography was performed using HiLoad™Superdex 75 pg 16/600 column (GE) and 50 mM Tris, 150 mM NaCl, pH 7.5 buffer for Angptl3 fibrinogen-like domain and 50 mM Tris, 200 mM NaCl, pH 7.4 buffer for Angptl4 fibrinogen-like domain. Fractions were analyzed on 12.5% SDS-PAGE and appropriate fractions combined and concentrated using Millipore Amicon® Stirred cell with 10 kDa cut-off membrane.

The small scale purification of mutant constructs of Angptl3 and Angptl4 was performed as described earlier^49^, except after the elution with 50 mM EDTA, 50 mM sodium phosphate buffer, pH 7.4, the proteins were buffer exchanged and concentrated into 50 mM Tris pH 7.5, 150 mM NaCl buffer using Amicon® Ultra -4 centrifugal concentrators with MWCO 10 000.

### Biophysical analysis

Solutions containing 10–40 μM protein and NEM-treated samples supplemented with 1:5 ratio of protein: 0.1% trifluoroacetic acid (TFA) were analyzed by Electrospray Ionization Mass Spectrometry (ESI-MS) (Synapt G2, Waters, Milford, MA, USA) coupled to Acquity Ultra Performance Liquid Chromatography (UPLC) machinery (Waters) with BEH300 C4 1.7 μm 2.1 × 10 mm UPLC column for desalting and protein separation prior to mass spectrometric analysis (Biocenter Oulu core facility). UPLC was performed using 0.4 ml/min flow with 0.1% formic acid and 3% to 70% acetonitrile gradient over 15 minutes.

### Thermal shift stability

The thermal shift stability of proteins was measured in 25 μl volume using 5 μg of protein and 5 × SYPRO Orange (Sigma-Aldrich) with 50 mM Tris pH 7.5, 150 mM NaCl. All measurements were performed in triplicates using Applied Biosystems 7500 Real Time PCR instrument, during which the protein samples were heated from 21 to 90 °C with 1 °C increment and the fluorescence was recorded. The melting temperature (T_m_) was calculated by examining of the derivative of rate of change versus temperature using IGOR (WaveMetrics Inc.).

### Protein crystallization

Crystallization screening was performed in the Biocenter Oulu core facility by the sitting-drop vapour-diffusion method in 96-well plates using TTP Labtech’s Mosquito LCP nanodispenser and commercially available sparse-matrix crystal screens, JCSG-*plus*™ and PACT *premier*™ (Molecular Dimensions). Multiple crystal hits were obtained for Angptl3 in JCSG-*plus*™ crystal screen. The best looking single crystals appeared in the condition containing 0.2 M Magnesium chloride hexahydrate, 0.1 M BIS-Tris pH 5.5, 25% (w/v) PEG 3350. Prior to data collection crystals, crystals were transferred into cryosolution containing 0.2 M Magnesium chloride hexahydrate, 0.1 M BIS-Tris pH 5.5, 35% (w/v) PEG 3350, briefly incubated and flash-frozen in liquid nitrogen. A single crystal of Angptl4 appeared in condition G12 of PACT *premier*™ HT-96 crystal screen (0.1 M Bis-Tris Propane pH 7.5, 0.2 M Sodium malonate, 20% (w/v) PEG 3350) in 2 weeks and was immediately transferred into cryosolution containing 0.1 M Bis-Tris Propane pH 7.5, 0.2 M Sodium malonate, 35% (w/v) PEG 3350, briefly incubated and flash-cooled in liquid nitrogen.

### Data collection

Crystal screening and X-ray diffraction data collection were performed at the European Synchrotron Radiation Facility (ESRF), Grenoble, France. High-resolution data for Angptl3 collected using *MASSIF-*1^50^, an automated high-throughput facility on beamline ID30a-1, at a wavelength of 0.966 Å and equipped with PILATUS3 2 M detector (DECTRIS). 105° of data were collected using 0.05° oscillation angle. High-resolution data for Angptl4 were collected from a single cryocooled crystal to a resolution of 2.3 Å on beamline ID29–1 equipped with Pilatus_6M detector (DECTRIS). 180° of data were collected at a wavelength of 0.9998 Å using a 0.15° oscillation angle. All data were processed using *XDS*^51^. Data collection statistics are presented in Table 1.

### Structure determination of the fibrinogen-like domains of Angptl3 and Angptl4

The crystal structures of the fibrinogen-like domain of Angptl3 and Angptl4 were determined by molecular replacement using Phaser-MR^52^ in the PHENIX software suite^53^ with previously determined structure of Angiopoietin 2 receptor binding domain (PDB code 1Z3S^41^) as the search model. Iterative rounds of model building and refinement were performed in Coot^54^ and phenix.refine^53^, respectively. In the earlier stages of refinement, 3-fold non-crystallographic symmetry restraints were utilized for Angptl3. In the later stages of refinement, atomic displacement parameters were also refined for both Angptl3 and Angptl4 in phenix.refine by TLS method^54^, and non-crystallographic symmetry restraints were released for Angptl3. Model geometry was monitored using MolProbity^55^. Figures were produced using UCSF Chimera^56^.

### Data availability

The structures generated during the current study are available in PDB under accession numbers 6EUA and 6EUB.

## Electronic supplementary material

Supplementary information
