# Supplementary material for: Structures of Angptl3 and Angptl4, modulators of triglyceride levels and coronary artery disease
Source: Sci Rep. 2018 Apr 30;8:6752. doi: 10.1038/s41598-018-25237-7 (PMC5928061; doi:10.1038/s41598-018-25237-7)
Supplement: Supplementary file 1 — Supplementary information [file 41598_2018_25237_MOESM1_ESM.pdf]

Supplementary information

Structures of Angptl3 and Angptl4, modulators of triglyceride levels and coronary artery disease

Ekaterina Biterova, Mariam Esmaeeli, Heli I. Alanen, Mirva Saaranen and Lloyd W. Ruddock

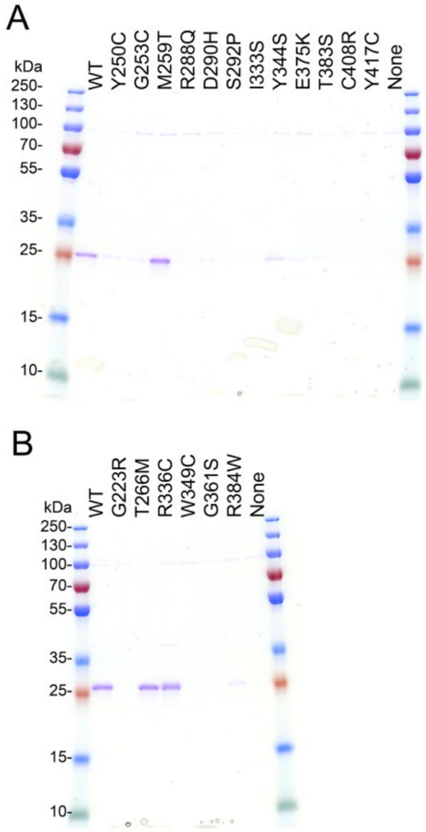

Supplementary figure 1: **Analysis of loss of function mutations.**

Coomassie stained SDS-PAGE gel of IMAC purified proteins. A Angptl3, B Angptl4.
